# Lack of Dependence of Indian Summer Monsoon Rainfall Extremes on Temperature: An Observational Evidence

**DOI:** 10.1038/srep31039

**Published:** 2016-08-03

**Authors:** H. Vittal, Subimal Ghosh, Subhankar Karmakar, Amey Pathak, Raghu Murtugudde

**Affiliations:** 1 Centre for Environmental Science and Engineering, Indian Institute of Technology Bombay, Mumbai 400 076, India; 2 Department of Civil Engineering, Indian Institute of Technology Bombay, Mumbai 400 076, India; 3 Interdisciplinary Program in Climate Studies, Indian Institute of Technology Bombay, Mumbai 400 076, India; 4 Earth System Science Interdisciplinary Centre (ESSIC)/DOAS, University of Maryland, College Park, Maryland, USA

## Abstract

The intensification of precipitation extremes in a warming world has been reported on a global scale and is traditionally explained with the Clausius-Clapeyron (C-C) relation. The relationship is observed to be valid in mid-latitudes; however, the debate persists in tropical monsoon regions, with the extremes of the Indian Summer Monsoon Rainfall (ISMR) being a prime example. Here, we present a comprehensive study on the dependence of ISMR extremes on both the 2 m surface air temperature over India and on the sea surface temperature over the tropical Indian Ocean. Remarkably, the ISMR extremes exhibit no significant association with temperature at either spatial scale: neither aggregated over the entire India/Tropical Indian Ocean area nor at the grid levels. We find that the theoretical C-C relation overestimates the positive changes in precipitation extremes, which is also reflected in the Coupled Model Intercomparison Project 5 (CMIP5) simulations. We emphasize that the changing patterns of extremes over the Indian subcontinent need a scientific re-evaluation, which is possible due to availability of the unique long-term *in-situ* data. This can aid bias correction of model projections of extremes whose value for climate adaptation can hardly be overemphasized, especially for the developing tropical countries.

The intensification of extreme precipitation events due to human-induced climate change is of great concern to society since it is a dominating factor in severe flooding and economic damage around the globe^1^,^2^. Despite the large internal variability in certain parts of the globe^3^, the increase in precipitation extremes is evident in some other parts of the globe due to a warming climate according to both observations^2^,^3^,^4^,^5^,^6^ and climate model simulations^7^,^8^. This increase in precipitation extremes in a warming world is due to the abundant availability of water vapor in a warmer atmosphere^9^,^10^,^11^. Studies based on numerical models^8^,^9^ indicate that the global-mean water vapor content increases at a rate of 7% per 1 K increase in surface temperature, which is approximately consistent with a constant effective relative humidity^10^. Precipitation extremes are expected to increase at the same rate or maybe even a higher rate if the strength of the updrafts associated with extreme precipitation events increases as the climate warms^9^,^12^. This dependence of precipitation extremes on temperature is considered to be thermodynamically determined. However, with respect to the theory, some studies using the observed datasets^13^,^14^ suggest that the increasing trend in precipitation extremes is not spatially uniform over the globe in a warming climate. It has also been observed that in some regions, specifically in the tropics, the rate of increase in precipitation extremes deviates from the C-C scaling, mainly due to the dynamic effects of the atmosphere^12^. In the tropics, the changes in precipitation extremes are thus largely determined by dynamic responses in the atmosphere rather than thermodynamic constraints^15^. In addition, a study conducted by Maeda *et al*.^16^ over the tropical region showed that the intensity of extreme precipitation is in fact decreasing with the increase in temperature and indicated that this relation may not be strongly influenced by the atmospheric moisture content on a daily time scale. Thus, the application of the theoretical C-C scaling in the tropics in predicting precipitation extremes still remains uncertain, and also the assessment of the relation between precipitation extremes and temperature continues to be poorly described^16^.

In India, being one of the major population centres and a developing tropical country with severe climate vulnerability, the response of precipitation extremes to the warming climate is of great concern for the water-food-energy nexus. Characteristics of extremes over India have changed dramatically in the recent past^17^,^18^,^19^,^20^ and there is considerable debate in attributing these changes to either global warming^21^,^22^ or local factors such as urbanization^23^. A reliable attribution of causes for the changing character of extremes is critical for the reliability of future projections for such resource-limited countries. Previous studies using observed datasets have noted an increase in the magnitude and frequency of extreme rainfall when spatially aggregated over central India and have attributed these changes to the warming environment^21^,^24^. However, follow-up research^25^,^26^,^27^,^28^ has indicated some major limitations associated with these studies. Furthermore, model-based analyses have shown an increase in the intensity of precipitation extremes in future projections^29^. On the other hand, it has been reported that the simulations of precipitation extremes in the current climate models are not reliable in the tropics^12^. In addition, the models also appear to misrepresent the trends in the ISMR^30^,^31^. Under these circumstances, a proper sensitivity evaluation of precipitation extremes due to warming over India is critical, especially in terms of refining the model projections of extremes when possible. In this context, plausibly for the first time in the reported literature, we have performed a comprehensive analysis of the dependence of the characteristics of ISMR extremes (rainfall from June to September) on temperature trends at a high resolution as afforded by the observed precipitation dataset (0.5° resolution). Please note that in the present study, the precipitation extreme refers to the heavy precipitation events. The changes in characteristics of ISMR extremes are examined by contrasting warm and cold years with respect to both sea surface temperature (SST) over the Tropical Indian Ocean and (TIO) land surface temperature over the Indian landmass (2AT). Furthermore, the results from the observed data have been compared with the CMIP5 models to assess the reliability of projections of precipitation extremes and their association with temperature.

## Results and Discussion

**Figure 1:**
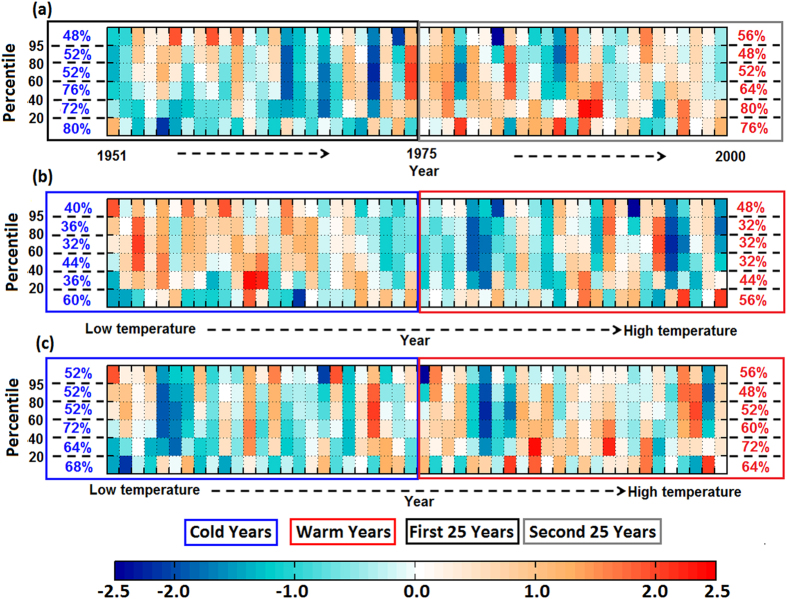
Anomalies of precipitation frequency in percentile bins of intensity based on chronological years. (**a**) Percentage of positive and negative anomaly rainfall years for different percentile category for pre- and post-1975 years. The lower percentile rainfall intensity shows an increase in frequency after 1975, though the signature of increase in extreme rainfall is not prominent for the higher percentile rainfall intensity. Further, extremes and its relation to 2AT (**b**) and SST in the TIO (**c**) are also examined. The percentage (%) values assigned on percentile bins show the percentage of years with negative (blue) and positive (red) anomalies during pre- to post-1975 and also during cold and warm years respectively, as defined by 2AT over India and TIO. The plots depict that the signature of increase in precipitation extremes with temperature is not significant. The figures were created using MATLAB (http://www.mathworks.com).

We first analyzed the pattern of an increase in ISMR extremes over India during the past 50 years. To accomplish this, grid-wise frequencies of different precipitation percentile categories (20pc, 20–40, 40–60, 60–80, 80–95 and >95pc) were computed and anomalies were spatially aggregated, which were then arranged on the basis of chronological years. Figure 1a shows the percentage of years with negative (blue) and positive (red) rainfall anomalies for different percentile categories before 1975 and after 1975. The pre- and post-1975 periods are chosen based on previous studies that have reported shifts in monsoon onset, withdrawal and the length of the monsoon season across 1975^32^,^33^. During pre-1975, the percentage of negative anomaly years dominates all the categories of precipitation frequencies, specifically for the lower percentiles. However, we note a dramatic change in the frequency of precipitation for the lower percentiles in the post-1975 period. Although an increase in frequencies of lower percentile precipitation is evident, the increase in extreme rainfall events is not so prominent for higher percentile rainfall intensity post-1975. The analysis was further carried out to find the changes in frequencies of different categories of precipitation in warming scenarios with respect to both the 2 m air temperature (2AT) spatially aggregated over India and the sea surface temperature (SST) over the Tropical Indian Ocean (TIO). Figure 1b depicts the anomalies in frequencies of different percentile categories of precipitation arranged according to cold and warm years of 2AT over India. In this case, the years are arranged from low to high based on their annual 2AT. The increase in the frequencies during the warm years is not prominent, especially in the case of the higher percentiles. Similarly, we observe that the increase in ISMR extremes is not prominent when the frequencies were arranged according to the years of low to high based on the annual SST over the TIO (Fig. 1c). We further plotted the PDFs and box plots of the low (0–20) and high (>95) percentiles to understand the changes in the precipitation of these percentile categories, both during pre- to post- 1975 and cold and warm years of 2AT and TIO respectively (Figure S3). We observe however that for the increase in low percentile precipitation categories both during pre- to post- 1975, the change in the distribution of high percentile precipitation is not statistically significant at 5% significance level during the warm years for either 2AT or TIO. In addition, we checked the association of the intensity of these extremes with both 2AT (average, minimum and maximum temperatures) and TIO, in terms of the spatial aggregate of the seasonal maximum (SM) precipitation (from June to September) all over India and the spatial mean of temperature. No significant dependence of SM precipitation is found on any category of temperature (Figure S4). This result is further supported by the correlation coefficients obtained from both parametric (Pearson) and nonparametric (Kendall’s Tau) tests (provided in each panel of Figure S4). Though, TIO is the main moisture source for the Indian summer monsoon rainfall (ISMR)^24^, there is an existence of substantial spatial variation in the amount of moisture diverge over this region. Based on the vertically integrated moisture flux (VIMF) and wind patterns over TIO obtained from ERA interim from 1979–2000, the major moisture divergence occurs from central and western part of TIO (Figure S5). Therefore, we divide the TIO into Central Indian Ocean (CIO), Western Indian Ocean (WIO) and Upper Indian Ocean (UIO) (Figure S6) and re-evaluate the dependence of ISMR extremes and SST over these regions. We carry out the analysis by spatially averaging the SM precipitation over 5 degree latitude bands and then estimate the correlation between these spatially aggregated precipitation extremes with the temperature considered across all the regions in the present study, including CIO, WIO and UIO from 1951–2000 (Figure S7). These results show a poor correlation between SM averaged precipitation across all the latitudes and temperature averaged over Indian land mass (2AT), and SSTs (TIO, CIO, WIO and UIO). It should be noted that the contribution of the moisture from these different moisture source regions may not be equally distributed over the Indian land mass, but may vary from region to region. For example, the moisture contribution from WIO to the Western Ghats and peninsular India will be higher (Figure S5), but it may decline towards Gangetic plains. Hence, here we divide the Indian landmass into three major sink regions, i.e., Ganga basin, south-central and peninsular Indian regions (Figure S8) and estimate the sensitivity of spatially aggregated SM precipitation extremes over these three regions with the temperature over different moisture sources. Result shows the precipitation extreme exhibit a poor correlation with the spatially averaged SSTs over CIO, WIO and UIO (Figure S9).

**Figure 2:**
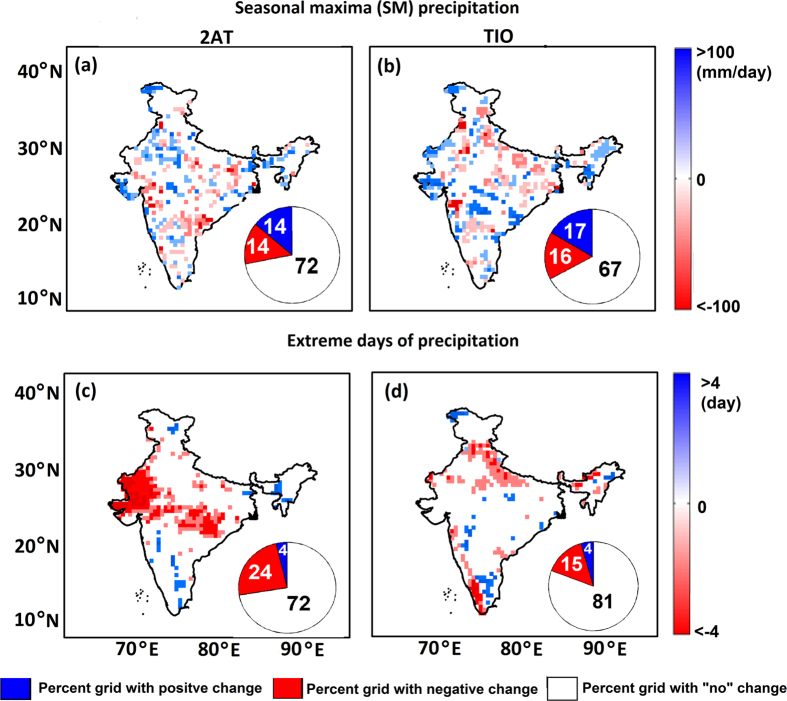
Changes in the 50-year RL of annual maximum intensity from cold to hot years for (**a**) 2AT and (**b**) SST. Following the bootstrap approach, the changes in RL are estimated at the 20% significance level. As shown, 72% and 67% of the grids show no significant changes in RL from cold to hot years for 2AT and TIO, respectively. The changes in the mean of extreme days from cold to hot years with respect to (**c**) 2AT and (**d**) TIO are examined at the 5% significance level. As shown, 72% and 81% of the grid points show no significant change in extreme days for 2AT and TIO, respectively. The maps are generated using ESRI ArcGIS 10.1 (http://www.esri.com/software/arcgis) and corresponding pie charts are created using MATLAB (http://www.mathworks.com).

The analysis on spatially aggregative ISMR extremes over India showed no significant association with temperature (Fig. 1 and Figure S4). However, precipitation extremes are a local phenomenon that displays a significant trend in spatial variability over India in the recent decades^28^. This motivated us to investigate the dependence of ISMR extremes on temperature at a relatively fine-resolution. Here, we evaluated the grid-wise changes in the characteristics of extreme precipitation such as SM intensity and extreme days, from cold to warm years, with respect to 2AT and TIO. The extreme days were identified by the implementation of a peak-over-threshold approach, with a threshold of the 95th percentile rainfall value^34^,^35^. In other words, extreme days were defined as the days during which rainfall events exceeded the 95th percentile threshold. The changes in the 50-year return level of SM intensities were estimated by the bootstrap approach^36^ (Figure S2) at a 20% significance level. The results show that only 28% and 33% of the grids have undergone significant changes from cold to warm years with respect to 2AT and TIO, respectively (Fig. 2a,b). Similarly, the analysis was performed for changes in the mean of extreme days at a 5% significance level. The results depict only 28% and 19% of the total grids exhibiting significant changes with respect to 2AT and TIO, respectively (Fig. 2c,d). In the case of extreme days, we observed some significant patterns in the changes. For instance, most of the grids with negative changes are found to appear in north-central India, while the changes were random in the case of SM intensity. We further extend our analysis by considering spatially aggregated temperature over CIO, WIO and UIO to define cold and warm years and perform the changes in the 50-year return level of SM intensities by the bootstrap approach (Figure S10). The results show that only 32%, 34% and 31% of the total grids have undergone significant changes from cold to warm years with respect to CIO, WIO and UIO, respectively. Overall our analysis suggested no prominent change in the characteristics of extreme precipitation from cold to warm years over India for either 2AT or SSTs over all the regions.

**Figure 3:**
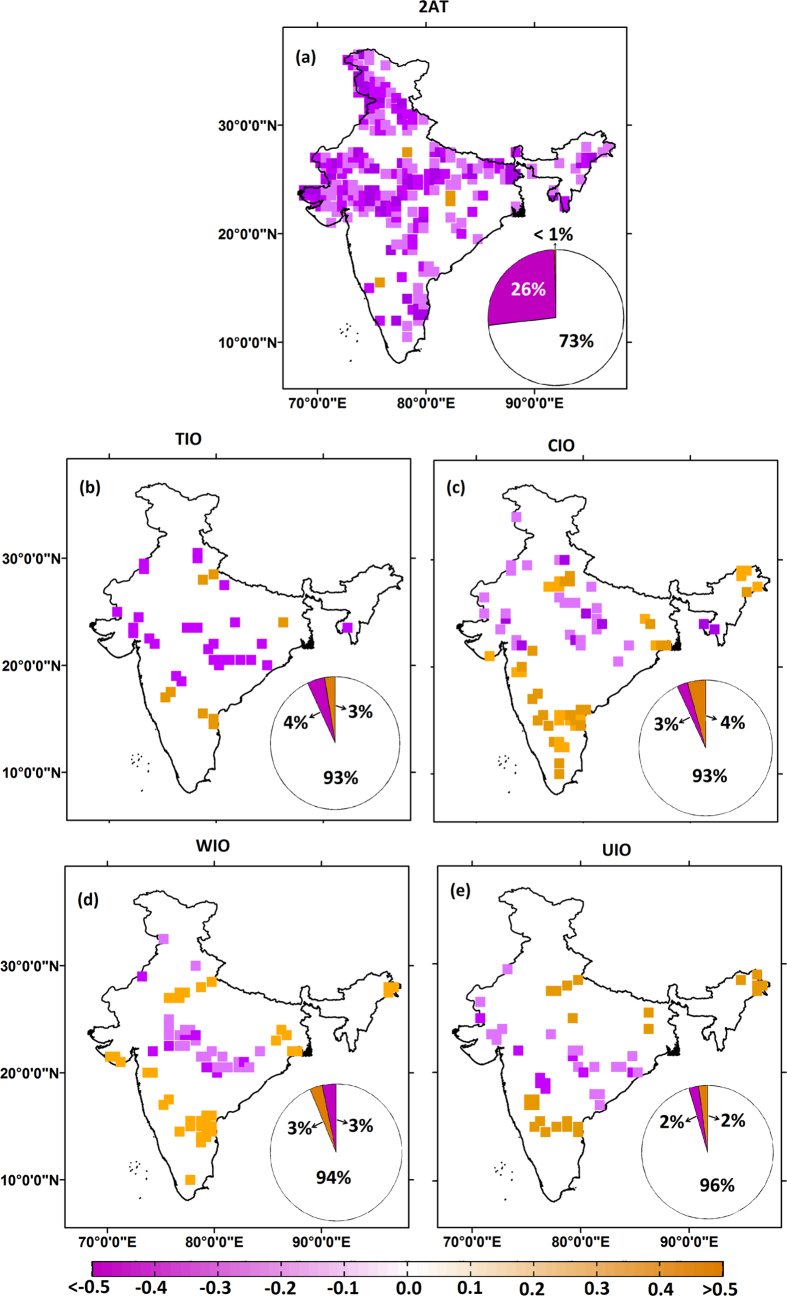
Correlation between SM precipitation extreme and temperature during the days of extreme events across all grid points over India. (**a**) Shows the correlation with respect to 2AT. The pie chart in each shows the percent of grids with significant positive, negative and insignificant correlation (at 5% significance level). Similarly (**b**–**e**) depict the correlation with respect to TIO, CIO, WIO and UIO. The maps are generated using ESRI ArcGIS 10.1 (http://www.esri.com/software/arcgis).

It is important to note that the analysis was performed by considering annual spatial averages of temperature over India to define hot and cold years. However, the precipitation extremes depend on the temperature at which they occur^12^. Therefore, we analyze the dependence of precipitation extreme and temperature, on the day of occurrence of extreme. Note that, in this case, the 2AT considered to evaluate the dependence is also at grid level, not spatially aggregated all over India. Here the analysis is performed from 1982–2000, since the daily SST data is only available starting from 1982. The Aphrodite temperature data is utilized here to analyze the dependence. Though the APHRODITE temperature data is available starting from 1960, we considered the data starting from 1982 to maintain the consistency with the daily SST data set. We observe that 26% of total grids exhibited significant negative correlation with 2AT, whereas 73% of the total grids show insignificant correlation with the temperature at 5% significance level (Fig. 3a). In case of SSTs, we observe 93%, 93%, 94% and 96% of total grids are having insignificant correlation with spatially averaged temperature over TIO (Fig. 3b), CIO (Fig. 3c), WIO (Fig. 3d) and UIO (Fig. 3e) respectively, on the day of extreme SM precipitation. Contrastingly, studies conducted by Wu *et al*.^37^ and Roxy^38^ have revealed the existence of a lead-lag relation between precipitation and temperature, specifically with SST. Keeping this in mind, we further evaluated the dependence between precipitation extremes and temperature on the lead-lag basis as well. Figure S11a shows the correlation of observed total precipitation with respect to lagged accumulated 2AT. We consider the lagged day, wherein the correlation is found to be maximum (either positive or negative) between these two variables. Here, the lags were considered up to 90 days, so that the pre-monsoon temperatures are also covered in the analysis. The analysis showed a positive correlation over most of the grids, especially at the west-coast and over northern parts of India. We also conducted a similar analysis by considering convective and stratiform precipitation, as the contributions of these two precipitation components to total precipitation tends to be almost equal over India^39^, and in addition, over mid-latitudes, these two precipitation components exhibit a positive association with temperature^40^. Figure S11b,c show an association between convective and stratiform precipitations with lagged accumulated 2AT, respectively. A similar pattern as in observed total precipitation is seen with most of the grids in the northern part of India exhibiting a positive correlation. However, negative correlations are prominent in the southern part of India for both convective and stratiform precipitations. We also carried out a similar analysis considering SM precipitation intensity. In this case, most grids have no significant correlation at the 5% significance level for total precipitation intensity (Figure S11d). The convective (Figure S11e) and stratiform precipitation (Figure S11f) components over India exhibit no prominent correlation with lagged 2AT either. A similar analysis was also performed by considering SST over the TIO. A dominance of significant negative correlations with lagged SST is obvious (Figure S11g) for total precipitation over India except for the Western Ghats and northeast regions of India. This result is consistent with Roxy *et al*.^41^, wherein they observed a negative relationship between the Indian Ocean SST and the ISMR. The analysis with convective (Figure S11h) and stratiform precipitation (Figure S11i) reveals a similar outcome as with total precipitation as well, i.e., most of the grids over the Indian landmass display significant negative correlations with SST. On the other hand, in the case of extremes, no significant correlation is observed in any of the three precipitation categories (Figure S11j–l). This finding firmly confirms that, although overall precipitation over India has a systematic relation with the lagged accumulated 2AT and SST, the extremes do not show any such association.

**Figure 4:**
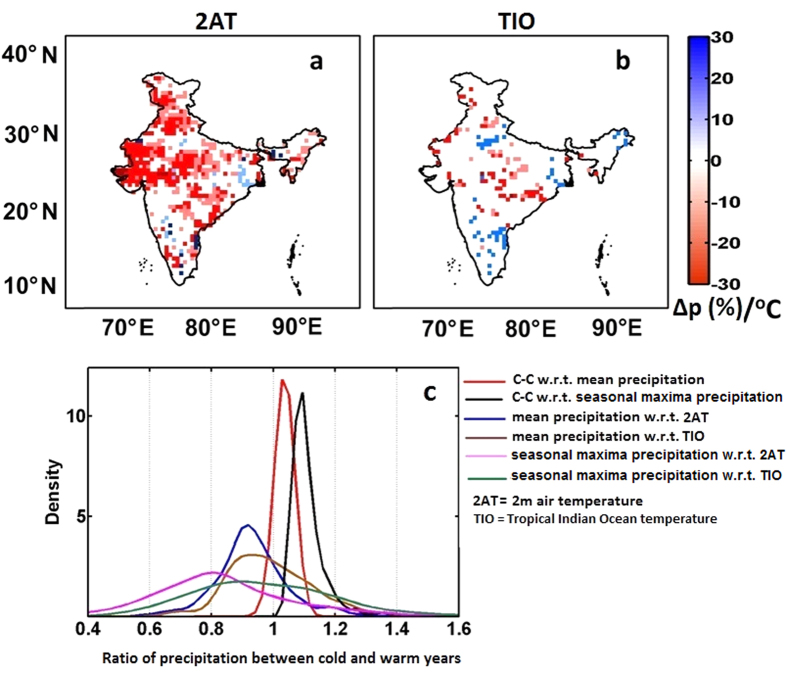
Changes in precipitation extremes (∆p expressed in %) for per degree increase in (**a**) 2AT and (**b**) TIO. The colored grids indicate a significant association between precipitation extremes and temperature. In the case of 2AT, most of the grids depict a negative change in the precipitation per degree increase in temperature; however, in the case of TIO, the maximum grids show no significant association. The changes in precipitation, both in its mean and extremes, are compared with the C-C scaling. (**c**) Here, the nonparametric kernel PDF of the ratio between hot to cold years by considering all the grids over India are plotted. The C-C scaling for both mean and extreme precipitation overestimates the changes when compared with the observed mean and extreme precipitation ratio. The maps are developed in ArcGIS 10.1 (http://www.esri.com/software/arcgis) and PDFs are generated using MATLAB (http://www.mathworks.com).

The physical explanation for the increase in the precipitation extremes with temperature is typically offered by the C-C scaling^42^. Contrastingly, Haerter *et al*.^43^ cautioned against the interpretation of the relation between these two variables with C-C scaling. This is because the tropical regions are known to exhibit changes in precipitation extremes that do not correspond to C-C scaling^6^,^16^. The present study computes the actual change in the precipitation extremes and compares them with the theoretical change, i.e., with C-C scaling. Initially, we quantify the changes in precipitation extremes with temperature (∆p/∆T) using an exponential regression, such that ∆p/∆T = 0.07 is equivalent to a C-C like scaling of 7% °C^−1^ at 25 °C^44^. In this case also, we have implemented 2AT at grid level from 1982–2000. Most of the grids with significant changes (36%) experience negative changes in precipitation extremes with respect to 2AT (Fig. 4a), and only a few grids undergo changes similar to or greater than the C-C scaling. Nonetheless, about 64% of the total grids exhibited insignificant change in the precipitation extremes. In the case of the TIO (Fig. 4b), the changes are not prominent since a significant number of grids show no significant changes at the 5% significance level. Our analysis reveals that the actual changes in precipitation extremes deviate from theoretical expectations, which was also reported by Utsumi *et al*.^6^. They found that a C-C like relation was dominant in the mid-latitudes but reported negative values of ∆p/∆T with respect to 2AT over the tropical region. This conclusion is also supported by Maeda *et al*.^16^ where a decrease in precipitation extremes with an increase in temperature was found over tropical regions of Brazil. We further compared the actual changes in the precipitation categories with the C-C scaling, as explained by Eq. 1. Here, we calculated the grid-wise ratio of precipitation categories (both mean and SM precipitations) between warm and cold years. A nonparametric kernel PDF was plotted considering all the grids over India (Fig. 4c). Warm and cold years in this case were obtained by arranging the years in an ascending order of temperature at each grid. The results show that the C-C scaling for SM and mean precipitation intensity have ratios >1. This result indicates that the theoretical response of precipitation extremes would be an increase during warm years. In contrast to this, the observed dataset shows that a majority of the grids have a ratio <1. This implies that the real observations do not support an intensification in either the mean or extreme precipitation over India in a warming world.

**Figure 5:**
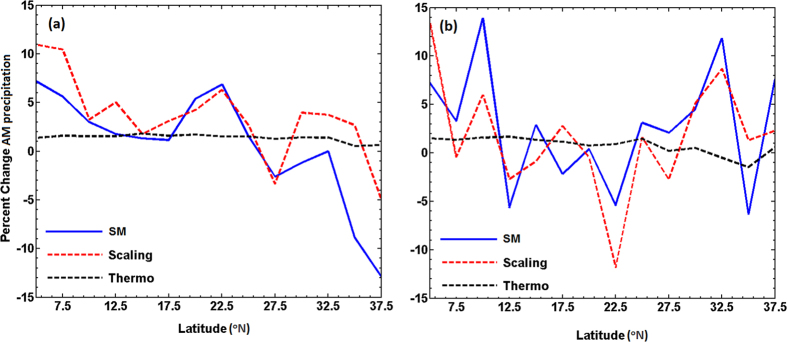
Changes in the SM precipitation, full precipitation extreme scaling (Eq. 1) and thermodynamic scaling for precipitation extreme from warm to cold years defined by surface temperature averaged over Indian region. (**a**) Shows the scaling for the NCEP1 reanalysis data from 1951–2000, whereas (**b**) is for ERA interim reanalysis dataset from 1979–2000. The figures were created using MATLAB (http://www.mathworks.com).

The changes in precipitation extremes can be understood by considering the dynamics and thermodynamics during the occurrence of extreme events^15^. Moreover, the C-C equation relates humidity with temperature and not necessarily precipitation extremes with temperature. Therefore, C-C scaling need not provide the correct scaling of the precipitation extreme with temperature. Considering these factors, we have evaluated the precipitation extreme scaling^12^, which contains both dynamic and thermodynamic components of the extremes. This will provide an insight on the major contributions from these two components to precipitation extremes. We utilize daily NCEP1 and ERA interim reanalysis temperature and vertical velocity from surface to 100 hpa pressure levels; and precipitation data at 2.5° resolution. We initially estimate precipitation extreme scaling at each grid and then the scaling is averaged over each latitude. Further, the changes in SM precipitation extremes and their scaling are estimated from warm to cold years based on the average surface temperature over entire India. We find that while the precipitation extreme scaling can capture the actual SM precipitation extreme behaviour at all the latitudes, the thermodynamic scaling (omitting ω_e_ term from Eq. 2) does not provide a good agreement with the actual SM precipitation extremes (Fig. 5). This observation is consistent with both NCEP1 (Fig. 5a) and ERA interim (Fig. 5b) reanalysis datasets. Our results indicate that the thermodynamic components have a minimal influence on changes in the characteristics of precipitation extremes, specifically over India.

In summary, our results indicate an insignificant increase in precipitation extremes with warming in 2AT or SSTs. The comparison with the theoretical expectation indicates that the C-C scaling overestimates the changes in mean as well as extreme precipitation with respect to temperature. This may be due to the fact that thermodynamic components (e.g., changes in the atmospheric moisture content) have a minimal influence on changes in the characteristics of precipitation extremes over India. The overall conclusion of the present study is in robust agreement with previous studies such as Emori and Brown^45^. The authors demonstrated that in the tropical regions, although dynamics (circulation changes) plays a secondary role, its influence on mean and extreme precipitation appears to be significant. This conclusion is further supported by Dairaku and Emori^15^, where the authors confined their study to the Asian summer monsoon. They concluded that the intensification of extreme precipitation over the South Asian land mass emerges from dynamic rather than thermodynamic changes. Moreover, during summer, moisture availability, and not the moisture holding capacity, plays a dominant role in the changing characteristics of precipitation extremes^46^.

**Figure 6:**
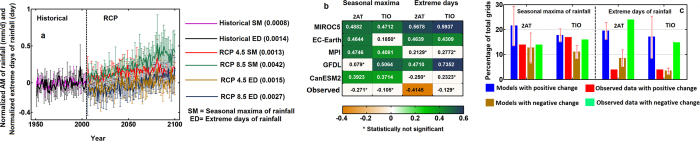
Comparison of the association between precipitation extremes and temperature with observed and CMIP5 models. (**a**) Shows the yearly variation in the multi-model means with error bars, of annual maximum precipitation and extreme days for both the historical period and RCP scenarios. The values inside the bracket represent the slope of the linear regression, suggesting that the trends in extremes will increase in the future. (**b**) Correlation matrix showing the correlation between spatial mean of annual maximum precipitation and extreme days with spatial mean of temperature from CMIP5 models and observed data over India. Most of the model extremes are positively correlated with both 2AT and TIO; however, the observed data show no significant correlation. The significant changes in the 50-year RL of annual maximum intensity (using the bootstrap approach) and changes in the mean of extreme days from cold to hot years (**c**) shows that models overestimate the significant changes in the characteristics of precipitation extremes. The figures are created in MATLAB (http://www.mathworks.com).

The analysis of observations thus reveals no significant association between the characteristics of extreme precipitation with either 2AT or SST. Understanding this process is foundational for predicting and projecting extremes since major decisions and policies are guided by these predictions and projections^47^. We evaluated the dependence of precipitation extremes on temperature in the CMIP5 model outputs. The multimodal mean of precipitation extremes during a historical period, i.e., from 1951–2005 shows an increasing trend. The magnitude of the trend was even higher for both SM precipitation and extreme days in the RCP 4.5 and 8.5 scenarios at a 5% significance level (Fig. 6a). Although the precipitation extremes in the models show an increase with warming scenarios, the analysis from the observed data reveals no such robust increase in extremes with warming. Hence, it is necessary to compute the association of ISMR extremes with temperature from the CMIP5 models and to compare it with the observations to understand the models’ biases and assess the reliability of their projections. Figure 6b depicts the matrix showing the correlations between the spatial mean of SM precipitation and extreme days with the spatial mean of temperature for the CMIP5 models and observations over India. The extremes in most of the models are significantly correlated with both 2AT and SST, which contradicts the observed behaviour. We also performed a grid-wise analysis of significant changes in the 50-year return level of AM (using the bootstrap approach) and significant changes in the mean of extreme days from cold to warm years (Fig. 6c). We observed that the percentage of grids with positive changes was relatively high in CMIP5 models in contrast to observations. In nature, the grids with negative change dominate for both the SM intensity and extreme days. This indicates that the models are overestimating precipitation extremes. Our results also have important implications for the need to quantify the association between intensification of precipitation extremes and temperature increase for the Indian subcontinent as well for other regions that are displaying detectable changes in extremes.

## Conclusions

Using 50 years of precipitation data, we have studied the complex relation between temperature and ISMR extremes over India. The physical explanation for the increase in precipitation extremes in a warming world is generally expected to be explained by the C-C equation. However, several studies^6^,^16^ have revealed that over tropical regions, the C-C relation does not necessarily offer a complete explanation. Goswami *et al*.^24^ and Rajeevan *et al*.^21^ reported increased precipitation extremes both in magnitude and frequency with a warming environment over India. However, these studies have some limitations^25^; for example, the analysis was on spatially aggregated quantities and the study region was assumed to be homogeneous, without performing any homogeneity tests. Therefore, a complete understanding of the association between precipitation extremes and temperature is clearly essential, especially for India, as the variation in precipitation has an immense influence on the water-food-energy nexus for the country. The present study focuses on such efforts and the following are the major conclusions:

1. The spatially aggregated and fine resolution analysis reveals an insignificant increase in extreme precipitation (intensity, extreme days and frequency), in response to either 2AT or SSTs. A lagged correlation analysis indicates the presence of a systematic association between overall precipitation and lagged accumulated temperature; however, we found its association with the precipitation extremes to be weak. This result from observations over India shows that there is no significant association between precipitation extremes and air or sea surface temperatures.
2. Changes in precipitation extremes (∆p expressed in %) per degree increase in 2AT and SST indicate that only a few grids experienced a change in extreme precipitation similar to or greater than that expected from C-C scaling, A majority of the grids underwent a negative change in response to warming. Furthermore, the comparison of actual change in the extremes and C-C scaling during warm and cold years points out that C-C scaling alone tends to overestimate the changes in both mean and extreme precipitation. Overall, this analysis indicates that the thermodynamic components have a minimal influence on the changes in the characteristics of precipitation extremes, specifically over India; however, the dynamic component has a significant influence^15^.
3. The CMIP5 models demonstrate an increase in both SM intensity and extreme days of precipitation under future warming scenarios. Nonetheless, the comparison with the observed data clearly demonstrates that the models are overestimating the association between extreme precipitation and temperature. As major decision-making and planning rely on the model predictions and projections, our results underscore the urgency for further research needed to resolve the temperature dependence of precipitation extremes.

These insights are clearly afforded by the availability of high-resolution precipitation data over India for a sufficiently long time. The challenge will clearly be daunting for regions where this luxury of observed data may not be in the offing. But the process insights gleaned over the Indian subcontinent should serve other regions as well in evaluating the models for their rendition of precipitation extremes. Considering the potential risks to the future of many developing countries, there is no choice to pursue these efforts with utmost seriousness and urgency.

## Methods

### Datasets used in the study

Gridded (0.5° resolution) daily rainfall data (mm/d) for 1950–2000 (50 years) during the monsoon months (June, July, August and September) over India were obtained from the Asian Precipitation-Highly Resolved Observed Data Integration Towards Evaluation of the Water Resource (APHRODITE) available for the entire Asia region^48^. The details regarding the data product are provided in Xie *et al*.^49^. The quality of the APHRODITE data is closely comparable to the data available from the India Meteorological Department (IMD) gridded rainfall data of 0.5° resolution^29^; both products capture the large-scale features of the Indian summer monsoon^50^. The 2 m air temperature (2AT) datasets over India (mean, maximum and minimum temperature) for the period of 1951–2000 were procured from CRU-CY (Climate Research Unit - Country wise dataset), which consists of monthly averages on a countrywide scale and are basically derived from the CRU dataset. The complete information pertaining to the data can be found at http://www.cru.uea.ac.uk/cru/data/hrg/ and are reported in Harris *et al*.^51^. The monthly sea surface temperature (SST) data from 1950–2000 for the Indian tropical ocean ranging from 24°S–24°N and 40°E–120°E (Figure S1) were obtained from the Hadley Centre Sea Ice and Sea Surface Temperature (HadISST) data set^52^. The SST zone for the present study was selected based on previous studies^24^,^53^, which showed its association with the variability of the ISMR.

The relationship between the SST and corresponding convective variables such as precipitation over the tropical monsoon region may not be instantaneous and may lag several days or even weeks^37^,^38^. Hence, in the present study, the correlation between precipitation extremes and lagged accumulated temperature has been examined for both 2AT and SST, with the utilization of daily datasets. The daily SST data was obtained from the NOAA High-resolution (0.25° resolution) Blended Analysis of Daily SST and Ice^54^ for the tropical Indian Ocean region. Daily 2AT was procured from APHRODITE from 1982–2000. Furthermore, it was identified that the contribution to total rainfall over India was equally shared by both convective and stratiform rainfall, varying within 45–55%^39^. Therefore, it is also important to analyze the dependence of both convective and stratiform precipitation extremes with temperature, along with total precipitation extremes. Thus, a lag correlation analysis was also performed for these two categories of rainfall over India. The convective and stratiform rainfall datasets were obtained from a high resolution (0.5° × 0.667°) reanalysis dataset, i.e., from the Modern-Era Retrospective analysis for Research and Application (MERRA) database^55^. The complete list of the data implemented in the present study is provided in Table S1. The results obtained from the observed data were then compared with the CMIP5 models to evaluate their performance in capturing the dependence between precipitation extremes and temperature. The details of the models used and their resolutions are provided in Table S2.

### Extraction of warm/cold years and the characterization of extremes

The cold and warm years were obtained with respect to the spatial mean of SST and 2AT, i.e., the spatial mean of 2AT (over India) and SST (over tropical Indian ocean) from 1951 to 2000 (50 years). The data were then arranged in ascending order. The corresponding years, with the lowest temperatures during the 25 years, were defined as “cold” years while “warm” years were those with the highest temperatures during the 25 years. The seasonal maximum (SM) precipitation was extracted for the cold and warm years, and the extremes were statistically modelled by implementing the extreme value theory (EVT). The Generalized Extreme Value (GEV) distribution, one type of EVT, was fitted for cold and warm SM intensity. The 50-year return level is computed from the Cumulative Distribution Function (CDF) and is defined as an extreme^28^,^56^. The goodness of fit test was estimated using the Kolomogorov-Smirnov (KS) test with a 5% significance level. Instead of GEV, a nonparametric kernel distribution^57^ was fitted to those grids where the AM intensity violated the KS test^17^. Furthermore, the changes in the 50-year return level (RL) between hot and cold years were estimated using the bootstrapping approach, following Kharin and Zwiers^36^, by sub-sampling (with repetition) the SM intensity 1000 times. These new samples were further used to re-estimate the 50-year RLs. The changes in the 50-year RLs between warm and cold years is statistically significant when their corresponding 60% confidence intervals (20th to 80th percentile) do not overlap, which approximately corresponds to a 20% statistical significance level. Figure S2 explains the step-by-step procedure involved in estimating the significant changes in the 50-year RL between warm and cold years.

### Physical relationship between precipitation extremes and temperature changes

The physics behind the relationship between precipitation extremes and temperature is argued to be explained by the C-C relation. The C-C relationship is typically expressed as a function of the saturation vapor pressure of water vapor with the atmospheric temperature, where the ratio of water vapor mass (or density, since the volume does not change) under different temperatures can be approximated based on the ideal gas law^58^.


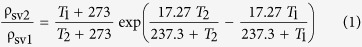


If the precipitation extremes are assumed to occur when the water vapor is nearly saturated and the amount of precipitation extremes is proportional to e_s_ (saturated vapor pressure), then the ratio ρ_sv1_/ρ_sv2_ provided in Eq. 1 represents the changes in precipitation mass (amount) from T_1_ (cold years temperature) to T_2_ (warm years temperature). Although the C-C relation explains the increase in precipitation extremes with an increase in temperature, it is unlikely to explain the same relation in tropical regions^16^. Drawing conclusions solely based on the C-C relation may thus be erroneous^43^,^45^. Hence, the present study compares the changes in extreme precipitation delineated from the observed data with the C-C scaling to verify whether the C-C relation explains the changes in precipitation extreme intensity over India under a warming scenario.

### Precipitation extreme scaling

The precipitation extreme scaling is evaluated using daily temperature and upward velocity at each pressure level conditioned on extreme precipitation occurring (here we consider SM) at the surface and is given by:


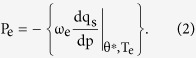


where, P_e_ is the SM precipitation, ω_e_ is the corresponding vertical velocity, {.} is a mass weighted integral over the troposphere. The moist-adiabatic derivative of saturation specific humidity is evaluated at the conditional mean temperature T_e_ when the SM precipitation occurs^12^. For the present study we utilize daily NCEP1^59^ and ERA interim^60^ reanalysis temperature and vertical velocity from surface to 100 hpa pressure levels; and precipitation data at 2.5 degree resolution.

### Estimation of correlation

The correlation between the precipitation extreme and temperature is estimated by implementation of Pearson correlation coefficient, which is given by the following equation:


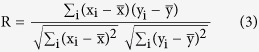


The p-value is estimated by implementing student t-test. If this probability is lower than the conventional 5% (P < 0.05) then correlation coefficient is called statistically significant.

## Additional Information

**How to cite this article**: Vittal, H. *et al*. Lack of Dependence of Indian Summer Monsoon Rainfall Extremes on Temperature: An Observational Evidence. *Sci. Rep*. **6**, 31039; doi: 10.1038/srep31039 (2016).

## Supplementary Material

Supplementary Information

## References

[b1] Pall P. . Anthropogenic greenhouse gas contribution to flood risk in England and Wales in autumn 2000. Nature 470(7334), 382–385 (2011). 21331040 10.1038/nature09762

[b2] Min S. K., Zhang X., Zwiers F. W. & Hegerl G. C. Human contribution to more-intense precipitation extremes Nature 470(7334), 378–381 (2011). 21331039 10.1038/nature09763

[b3] Deser C., Knutti R., Solomon S. & Phillips A. S. Communication of the role of natural variability in future North American climate. Nature Climate Change 2 **(11)**, 775–779 (2012).

[b4] Allan R. P., Soden B. J., John V. O., Ingram W. & Good P. Current changes in tropical precipitation. Environ. Res. Lett. 5, 025205 (2010).

[b5] Liu S. C., Fu C., Shiu C., Chen J. & Wu F. Temperature dependence of global precipitation extremes. Geophys. Res. Lett. 36, L17702 (2009).

[b6] Utsumi N., Seto S., Kanae S., Maeda E. E. & Oki T. Does higher surface temperature intensify extreme precipitation? Geophys. Res. Lett 38, LI6708 (2011).

[b7] Meehl G. A. . An introduction to trends in extreme weather and climate events: observations, socioeconomic impacts, terrestrial ecological impacts, and model projections. Bulletin of the American Meteorological Society 81 **(3)**, 413–416 (2000).

[b8] Kharin V. V., Zwiers F. W., Zhang X. & Hegerl G. C. Changes in temperature and precipitation extremes in the IPCC ensemble of global coupled model simulations. J. Climate 20 **(8)**, 1419–1444 (2007).

[b9] Allen M. R. & Ingram W. J. Constraints on future changes in climate and the hydrologic cycle. Nature 419, 224–232 (2002). 12226677 10.1038/nature01092

[b10] Held I. M. & Soden B. J. Robust responses of the hydrological cycle to global warming. J. Climate 19, 5686–5699 (2006).

[b11] Muller C., O’Gorman P. A. & Back L. E. Intensification of precipitation extremes with warming in a cloud-resolved model. J. Climate 24, 2784–2800 (2011).

[b12] O’Gorman P. A. & Schneider T. The physical basis for increase in precipitation extremes in simulations of 21st-century climate change. Proc. Natl. Acad. Sci. USA 106, 14773–14777 (2009). 19706430 10.1073/pnas.0907610106 PMC2736420

[b13] Groisman P. Y. . Trends in intense precipitation in the climate record. J. Clim 18, 1326–1350 (2005).

[b14] Alexander L. V. . Global observed changes in daily climate extremes of temperature and precipitation. J. Geophys. Res. 111, D05109 (2006).

[b15] Dairaku K. & Emori S. Dynamic and thermodynamic influences on intensified daily rainfall during the Asian summer monsoon under doubled atmospheric CO2 conditions. Geophys. Res. Lett. 33, L01704 (2006).

[b16] Maeda E. E., Utsumi N. & Oki T. Decreasing precipitation extremes at higher temperatures in tropical regions. Nat. Hazards 64, 935–941 (2012).

[b17] Vittal H., Karmakar S. & Ghosh S. Diametric changes in trends and patterns of extreme rainfall over India from pre-1950 to post-1950. Geophys. Res. Lett. 40, 3253–3258 (2013).

[b18] Dash S. K., Kulkarni M. A., Mohanty U. C. & Prasad K. Changes in the characteristics of rain events in India. J. Geophys. Res. 114, D10109, doi: 10.1029/2008JD010572 (2009).

[b19] Singh D., Tsiang M., Rajaratnam B. & Diffenbaugh N. S. Observed changes in extreme wet and dry spells during the South Asian summer monsoon season. Nature Climate Change 4 **(6)**, 456–461(2014).

[b20] Vinnarasi R. & Dhanya C. T. Changing characteristics of extreme wet and dry spells of Indian monsoon rainfall. J. Geophys. Res, doi: 10.1002/2015JD024310 (2016).

[b21] Rajeevan M., Bhate J. & Jaswal A. K. Analysis of variability and trends of extreme rainfall events over India using 104 years of gridded daily rainfall data. Geophys. Res. Lett 35 **(18)**, L18707, doi: 10.1029/2008GL035143 (2008).

[b22] Mani N. J., Suhas E. & Goswami B. N. Can global warming make Indian monsoon weather less predictable? Geophys. Res. Lett 36 **(8)**, doi: 10.1029/2009GL037989 (2009).

[b23] Kishtawal C. M., Niyogi D., Tewari M., Pielke R. A. & Shepherd J. M. Urbanization signature in the observed heavy rainfall climatology over India. International Journal of Climatology 30 **(13)**, 1908–1916 (2010).

[b24] Goswami B. N., Venugopal V., Sengupta D., Madhusoodanan M. S. & Xavier P. K. Increasing trend of extreme rain events over India in a warming environment. Science 314, 1442–1445 (2006). 17138899 10.1126/science.1132027

[b25] Ghosh S., Luniya V. & Gupta A. Trend analysis of Indian summer monsoon rainfall at different spatial scales. Atmos. Sci. Let. 10, 285–290 (2009).

[b26] Krishnamurthy C. K. B., Lall U. & Kwon H. H. Changing frequency and intensity of rainfall extremes over India from 1951–2003. J. Clim. 22, 4737–4746 (2009).

[b27] Satyanarayana P. & Srinivas V. V. Regional frequency analysis of precipitation using large-scale atmospheric variables. J. Geophys. Res. 113, D24110 (2008).

[b28] Ghosh S., Das D., Kao S. C. & Ganguly A. R. Lack of uniformtrends but increasing spatial variability in observed Indian rainfall extremes. Nat. Clim. Change 2 **(2)**, 86–91 (2012).

[b29] Rajendran K., Kitoh A., Srinivasan J., Mizuta R. & Krishnan R. Monsoon circulation interaction with Western Ghats orograpy under changing climate. Theor. App. Climatol 110, 555–571 (2012).

[b30] Saha A., Ghosh S., Sahana A. S. & Rao E. P. Failure of CMIP5 climate models in simulating post‐1950 decreasing trend of Indian monsoon. Geophysical Research Letters 41 **(20)**, 7323–7330 (2014).

[b31] Sabeerali C. T., Rao S. A., Dhakate A. R., Salunke K. & Goswami B. N. Why ensemble mean projection of south Asian monsoon rainfall by CMIP5 models is not reliable? Climate Dynamics 45 **(1–2)**, 161–174 (2015).

[b32] Sabeerali C. T. . Modulation of monsoon intraseasonal oscillations in the recent warming period. Journal of Geophysical Research: Atmospheres 119 **(9)**, 5185–5203 (2014).

[b33] Sahana A. S., Ghosh S., Ganguly A. & Murtugudde R. Shift in Indian summer monsoon onset during 1976/1977. Environmental Research Letters 10 **(5)**, 054006 (2015).

[b34] Haylock M. & Nicholls N. Trends in extreme rainfall indices for an updated high quality data set for Australia, 1910-1998. Int. J. Climatol. 20, 1533–1541 (2000).

[b35] Khan S., Kuhn G., Ganguly A. R., Erickson D. J. & Ostrouchov G. Spatio-temporal variability of daily and weekly precipitation extremes in South America. Water Resour. Res. 43, W11424 (2007).

[b36] Kharin V. V. & Zwiers F. W. Estimating extremes in transient climate change simulations. J. Clim. 18, 1156–1173 (2005).

[b37] Wu R., Kirtman B. P. & Pegion K. Local rainfall-SST relationship on sub seasonal time scales in satellite observations and CFS. Geophys. Res. Lett. 35, L22706 (2008).

[b38] Roxy M. K. Sensitivity of precipitation to sea surface temperature over the tropical summer monsoon region-and its quantification. Clim. Dyn, doi: 10.1007/s00382-013-1881-y (2013).

[b39] Pokhrel S. & Sikka D. R. Variability of the TRMM-PR total and convective and stratiform rain fractions over the Indian region during the summer monsoon. Clim. Dyn 41, 21–44 (2013).

[b40] Berg P., Moseley C. & Haerter J. O. Strong increase in convective precipitation in response to higher temperatures. Nat. Geosci. 6, 181–185 (2013).

[b41] Roxy M. K. . Drying of Indian subcontinent by rapid Indian Ocean warming and a weakening land-sea thermal gradient. Nature communications 6, doi: 10.1038/ncomms8423 (2015). 26077934

[b42] Trenberth K. E., Dai A., Rasmussen R. M. & Parsons D. B. The changing character of precipitation. Bull. Am. Meteorol. Soc. 84, 1205–1217 (2003).

[b43] Haerter J. O., Berg P. & Hagemann S. Heavy rain intensity distributions on varying time scales and at different temperatures. J. Geophys. Res. 115, D17102 (2010).

[b44] Jones R. H., Westra S. & Sharma A. Observed relationships between extreme sub-daily precipitation, surface temperature and relative humidity. Geophys. Res. Lett. 37, L22805 (2010).

[b45] Emori S. & Brown S. J. Dynamic and thermodynamic changes in mean and extreme precipitation under changed climate. Geophys. Res. Lett 32, L17706 (2005)

[b46] Berg P. . Seasonal characteristics of the relationship between daily precipitation intensity and surface temperature. J. Geophys. Res. 114, D18102 (2009).

[b47] Villegas J. R. & Challinor A. Assessing relevant climate data for agricultural applications. Agricultural and Forest Meteorology 161, 26–45 (2012).

[b48] Yatagai A. . APHRODITE: Constructing a Long-term Daily Gridded Precipitation Dataset for Asia based on a Dense Network of Rain Gauges. BAMS, doi: 10.1175/BAMS-D-11-00122.1 (2005).

[b49] Xie P. . A gauge-based analysis of daily precipitation over East Asia. Journal of Hydrometeorology 8, 607–627 (2007).

[b50] Rajeevan M. & Bhate J. A high resolution daily gridded rainfall dataset (1971–2005) for mesoscale meteorological studies. Curr. Sci 96(4), 558–562 (2009).

[b51] Harries I., Jones P. D., Osborn T. J. & Lister D. H. Updated high-resolution grids of monthly climate observations- the CRU TS3.10 Dataset. Int. J. Climatol. 34, 623–642 (2014).

[b52] Rayner N. A. . Global analyses of sea surface temperature, sea ice, and night marine air temperature since the late nineteenth century. J. Geophys. Res. 108, doi: 10.1029/2002JD002670 (2003).

[b53] Clark C. O., Cole J. E. & Webster P. J. Indian ocean SST and Indian summer rainfall: predictive relationships and their decadal variability. J. Clim. 13, 2503–2519 (1999).

[b54] Reynolds R. W. . Daily high-resolution-blended analyses for sea surface temperature. J. Clim. 20, 5473–5496 (2007).

[b55] Rienecker . MERRA: NASA’s modern-era retrospective analysis for research and applications. J. Clim. 24, 3624–3648 (2011). 10.1175/JCLI-D-16-0758.1 PMC6999672 32020988

[b56] Coles S. G. An introduction to statistical modeling of extreme values, London, Springer (2001).

[b57] Bowman A. & Azzalini A. Applied smoothing techniques for data analysis: the kernel approach with S-plus illustrations, Oxford Univ. Press, New York (1997).

[b58] Kao S. C. & Ganguly A. R. Intensity, duration, and frequency of precipitation extremes under 21st-century warming scenarios. J. Geophys. Res. 116, D16119 (2011).

[b59] Kalnay E. . The NCEP/NCAR 40-year reanalysis project. BAMS 77 **(3)**, 437–471 (1996).

[b60] Berrisford P. . The ERA-Interim archive Version 2.0, ERA Report Series 1, ECMWF. Shinfield Park. Reading, UK, 13177 (2011).

