# Supplementary material for: Lack of Dependence of Indian Summer Monsoon Rainfall Extremes on Temperature: An Observational Evidence
Source: Sci Rep. 2016 Aug 3;6:31039. doi: 10.1038/srep31039 (PMC4971573; doi:10.1038/srep31039)
Supplement: Supplementary Information [file srep31039-s1.doc]

***Supplementary Information for***

**Lack of Dependence of Indian Summer Monsoon Rainfall Extremes on Temperature: An Observational Evidence**

Vittal, H.^1^ , Subimal Ghosh^2,3^, Subhankar Karmakar^1,3*^, Amey Pathak^2^

and Raghu Murtugudde^3,4^

^1^ Centre for Environmental Science and Engineering, Indian Institute of Technology Bombay, Mumbai 400 076, India.

^2^ Department of Civil Engineering, Indian Institute of Technology Bombay,

Mumbai 400 076, India.

^3^ Interdisciplinary Program in Climate Studies, Indian Institute of Technology Bombay, Mumbai 400 076, India.

^4^Earth System Science Interdisciplinary Centre (ESSIC)/DOAS, University of Maryland, College Park, Maryland, USA.

*Corresponding Author

E-mail: skarmakar@iitb.ac.in

Phone: +91 22 2576 7857

**Supplementary Figures and Tables**


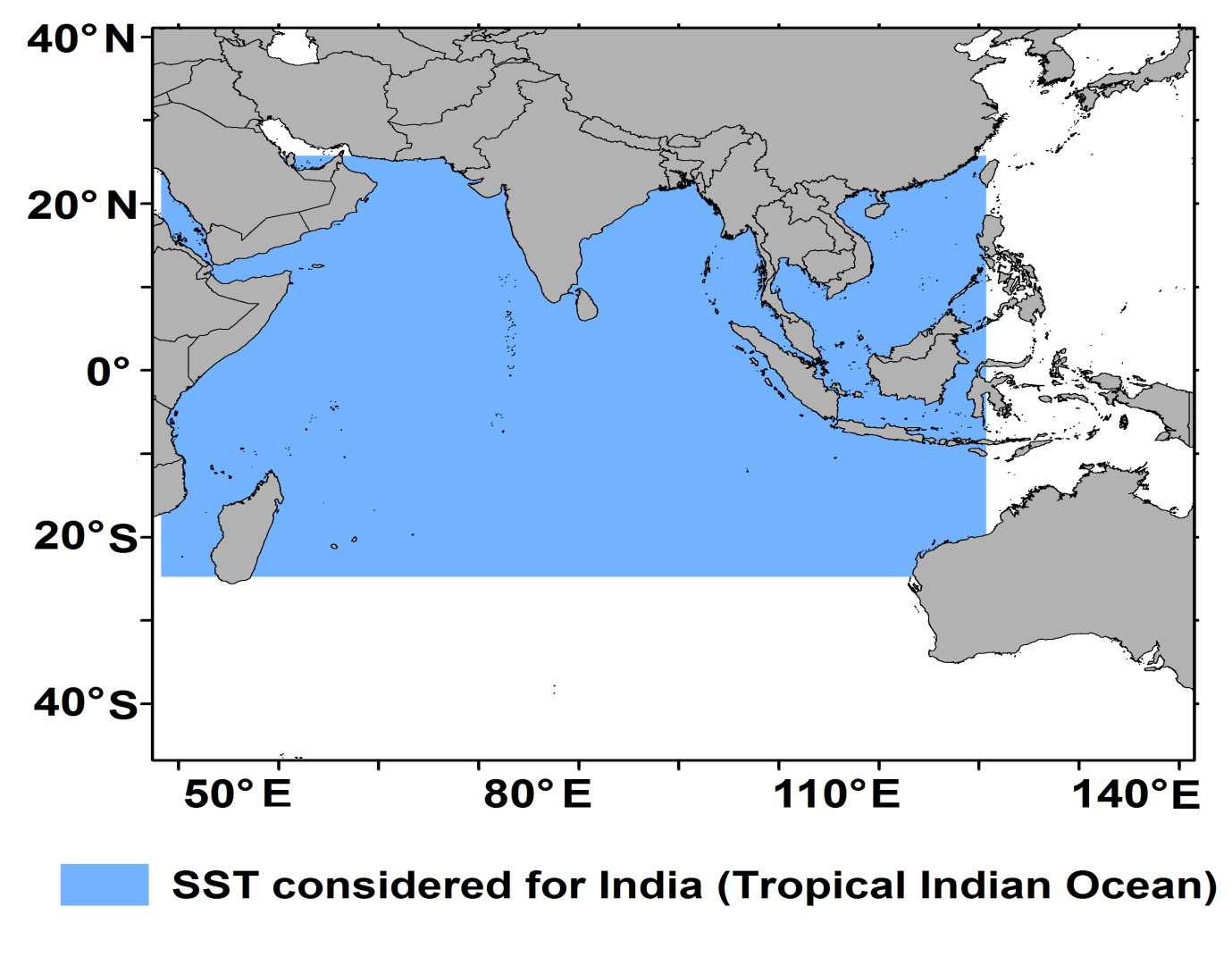


**Figure S1**: The region considered for sea surface temperature (SST) over the Tropical Indian Ocean (TIO). The map is generated using ArcGIS 10.1 (http://www.esri.com/software/arcgis).


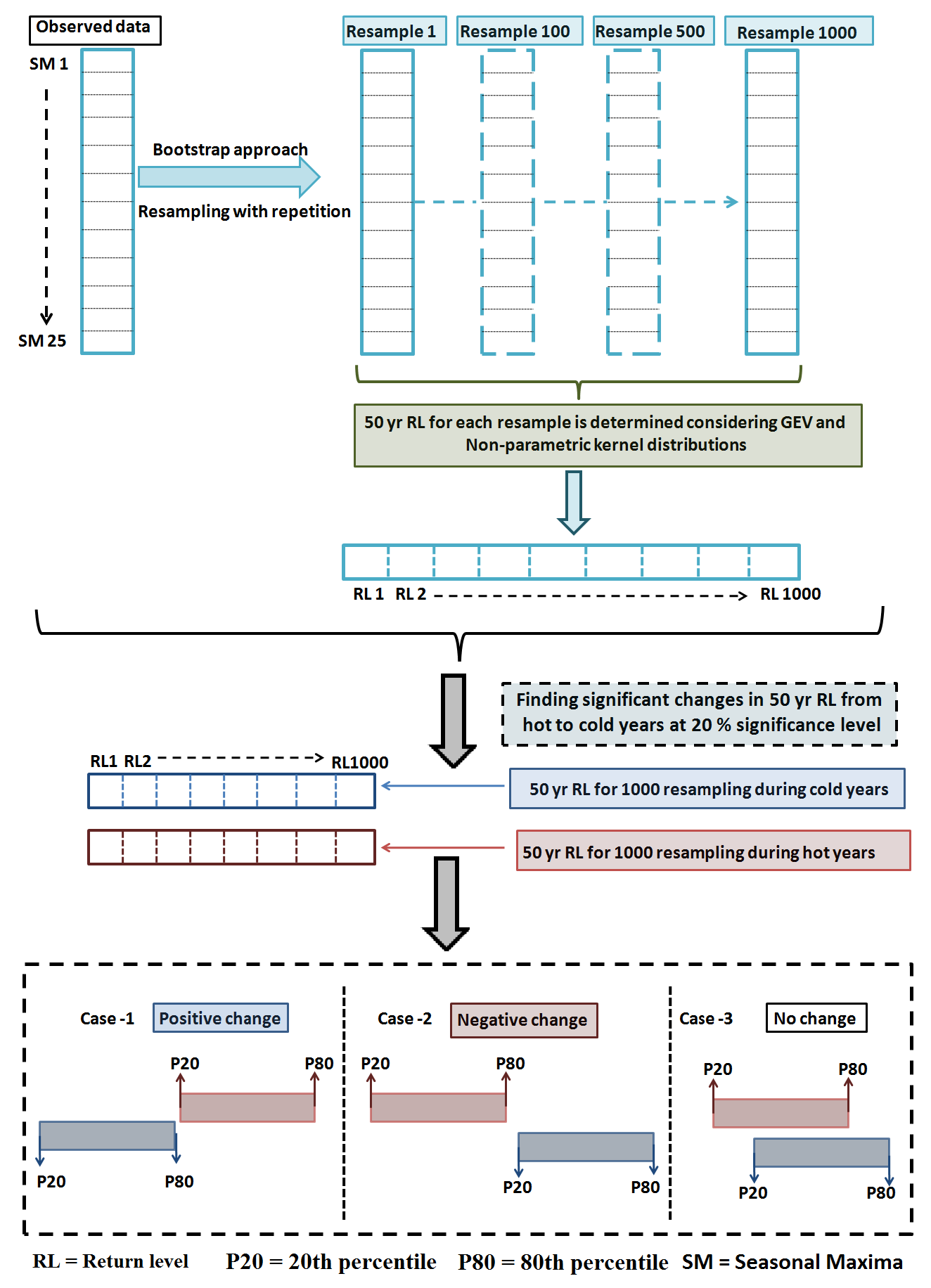


**Figure S2:** A schematic diagram of the bootstrapping approach.

**
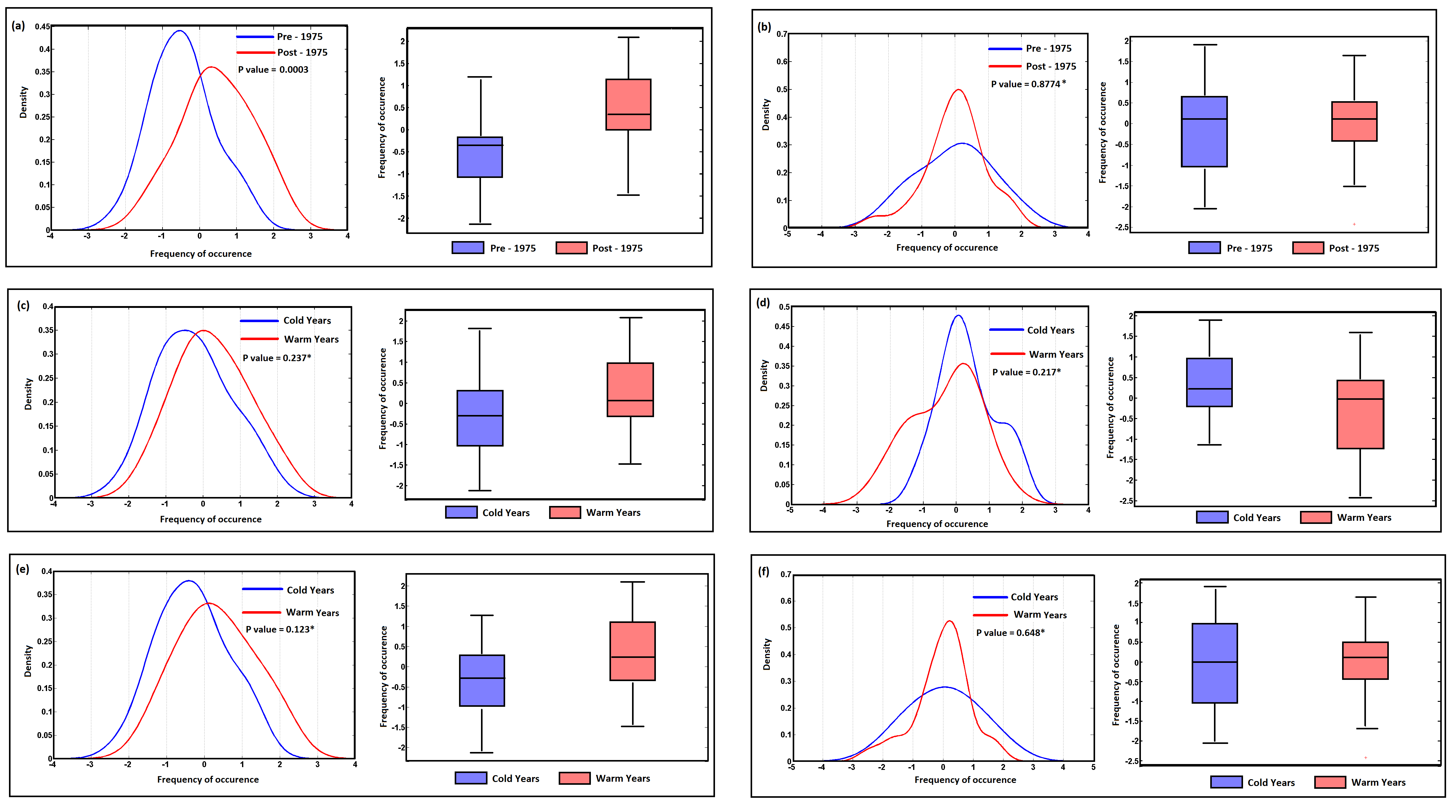
**

**Figure S3:** PDFs and box plots showing the changes in the distribution of frequency of occurrence of low and high percentiles of the precipitation. (a) Shows the changes in the distribution of low percentile precipitation, whereas (b) depicts the changes in the distribution of the high percentile precipitation distribution during pre- to - post 1975. (c) and (d) shows the changes in PDFs and box plots for low and high percentiles of the precipitation during cold to warm years as defined by 2AT respectively. (e) and (f) is same as (c) and (d) but for SST over TIO.P values from the KS test are shown in in each respective figure.. Here, we observe that apart from (a) all others show insignificant changes in the PDFs at 5% significance level. The figures are developed using MATLAB (http://www.mathworks.com).


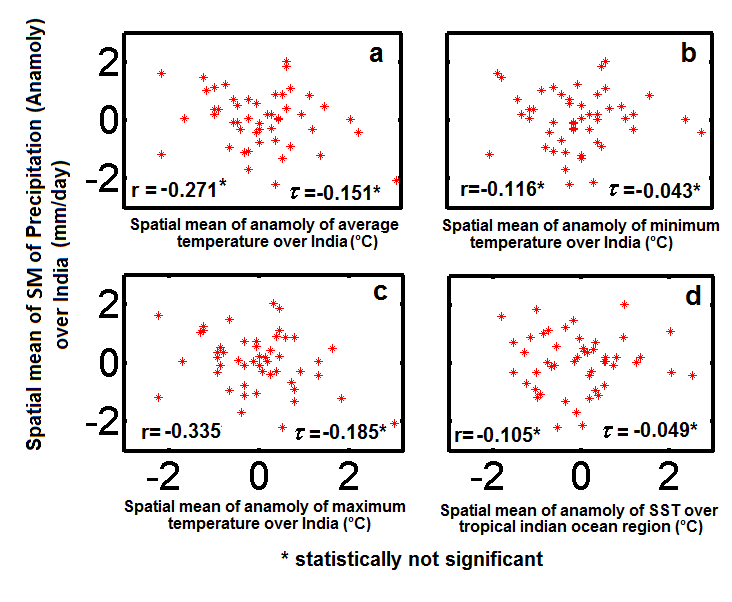


**Figure S4:** Scatter plots of the anomaly of spatial mean of seasonal maximum (SM) precipitation over India with respect to spatial mean of (a) anomaly of average temperature over India (b) anomaly of minimum temperature over India (c) anomaly of maximum temperature over India and (d) anomaly of SST over tropical Indian Ocean. These plots show no significant dependence of seasonal precipitation maxima on temperature. The values inside the box represent Pearson and Kendall's Tau correlation coefficients, which also reveals no significant association between precipitation extremes and temperature. The figures are developed using MATLAB (http://www.mathworks.com).


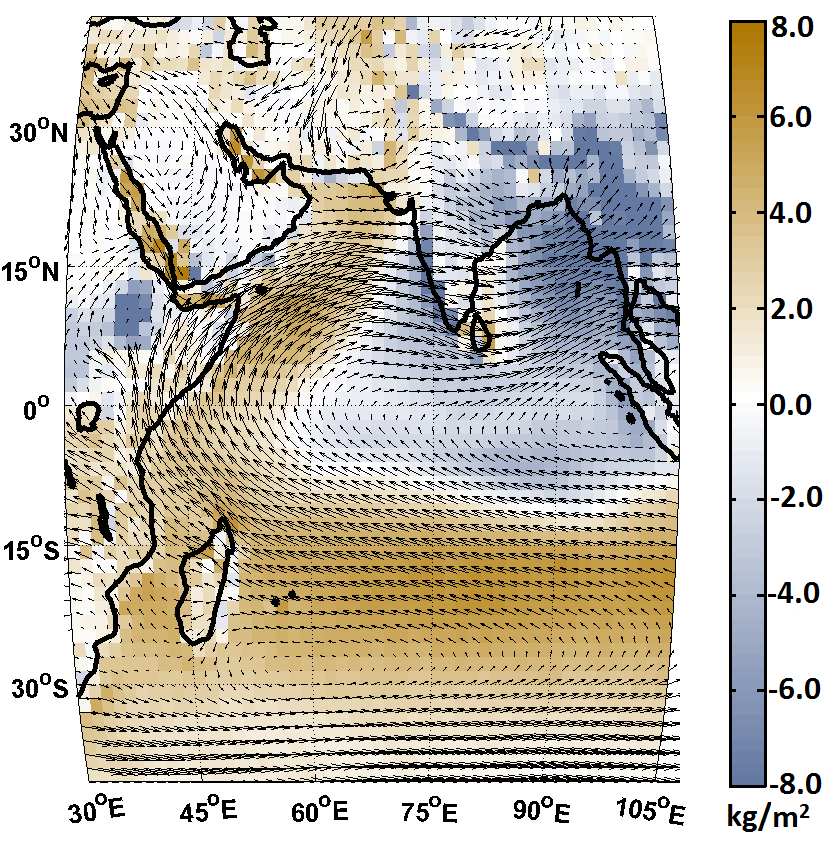


**Figure S5:** Vertically integrated moisture flux (VIMF) and wind patterns over India for monsoon season during 1979-2000. The positive (negative) value of VIMF exhibits divergence (convergence) over the study region. The figures are developed using MATLAB (http://www.mathworks.com).


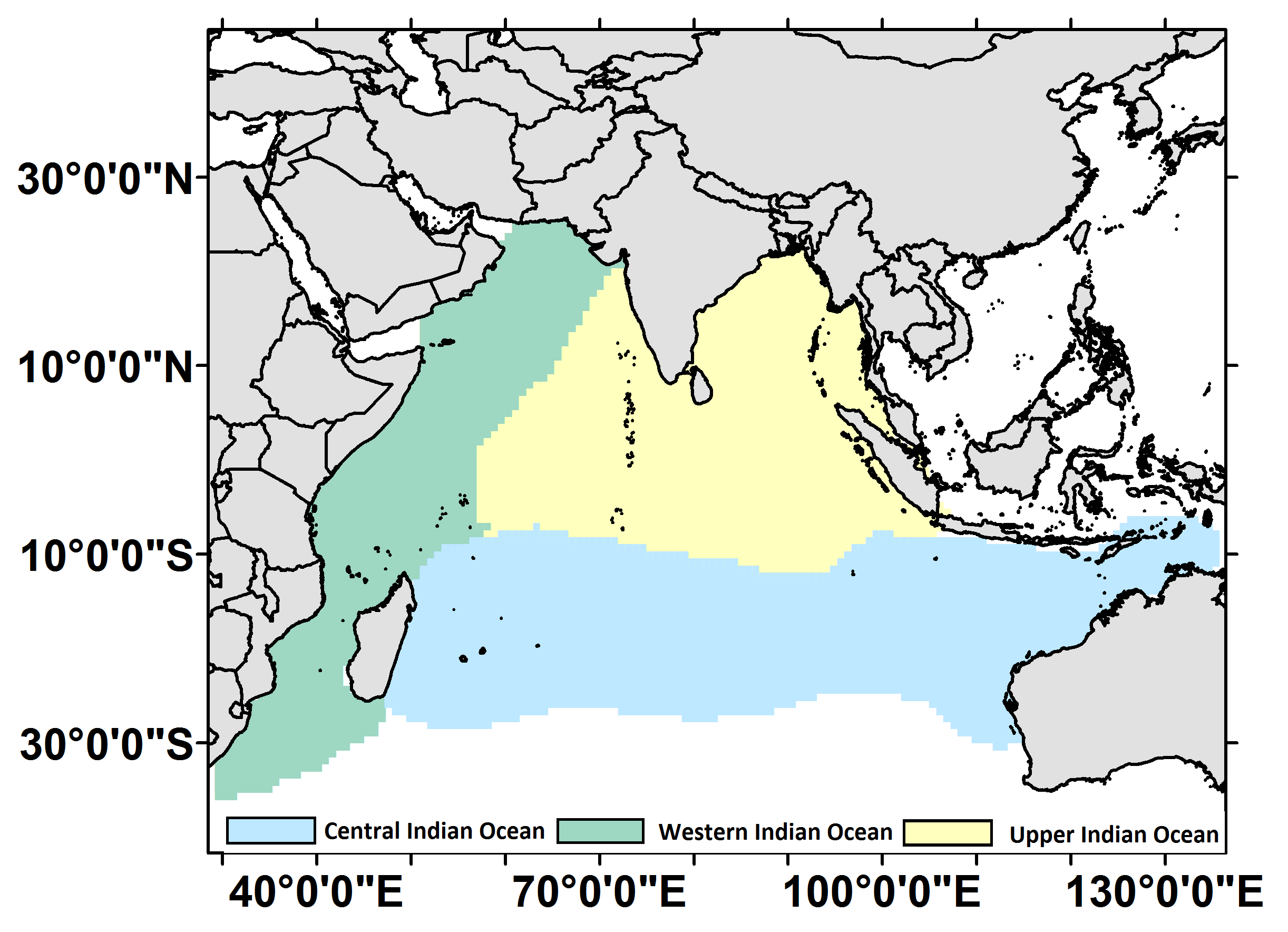


**Figure S6:** Different major moisture sources for Indian summer monsoon rainfall (ISMR). The map is generated using ArcGIS 10.1 (http://www.esri.com/software/arcgis).


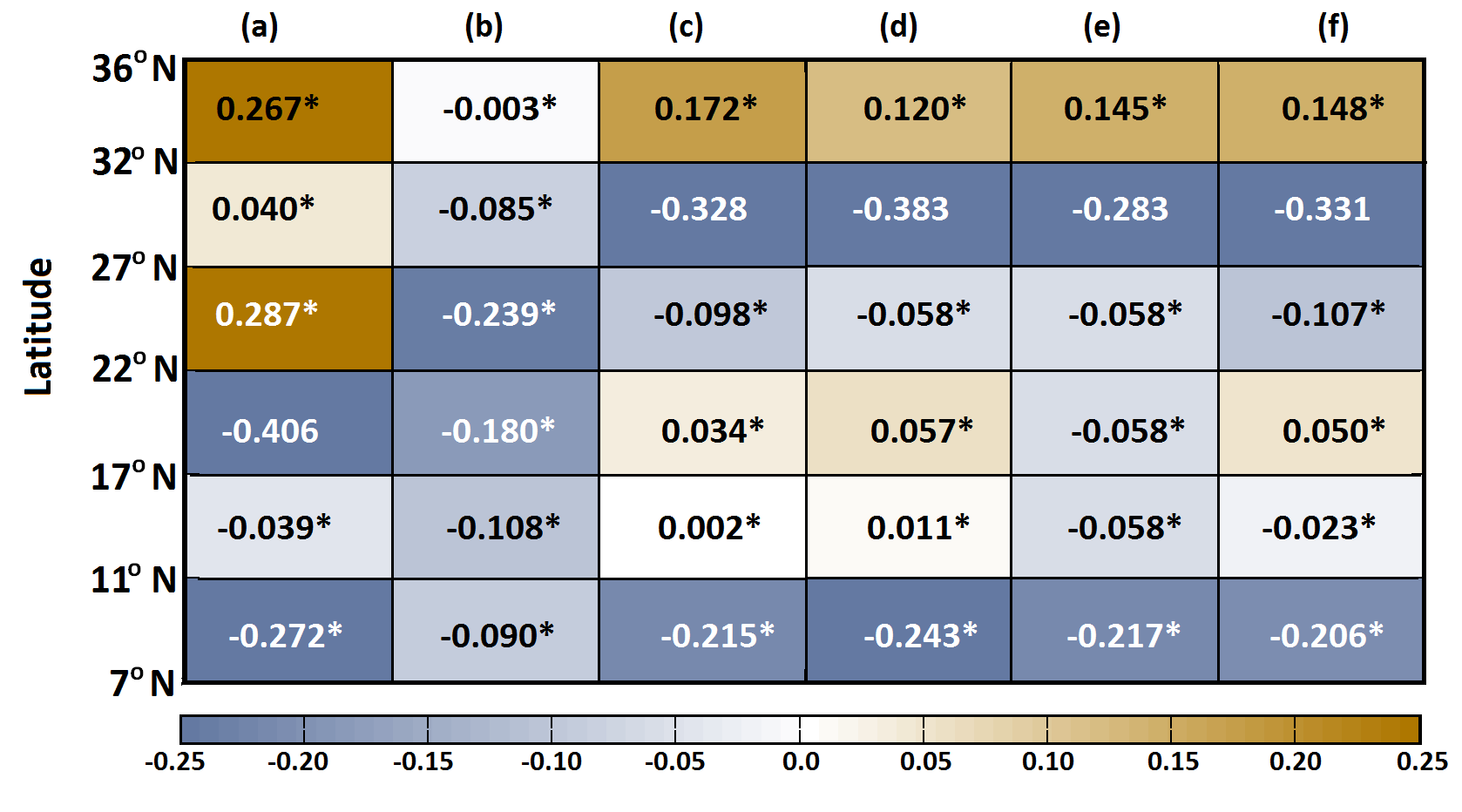


**Figure S7:** Correlation between SM precipitation extreme which is spatially aggregated across 5 degree latitude band and different categories of temperature. (a) shows the correlation with the gridded 2AT, which are also spatially averaged across 5 degree latitude band over India from 1982-2000. (b) depicts the correlation with the spatially aggregated 2AT all over India (2AT) from 1951-2000. (c) shows the correlation with the tropical Indian Ocean (TIO), whereas (d), (e) and (f) are Central Indian Ocean (CIO), Western Indian Ocean (WIO) and Upper Indian Ocean (UIO) from 1951-2000, respectively. The values inside the boxes represents the magnitude of the Pearson correlation and (*) indicates the correlation between SM precipitation and temperature is statistically insignificant at 5% significance level. The figures are developed using MATLAB (http://www.mathworks.com).


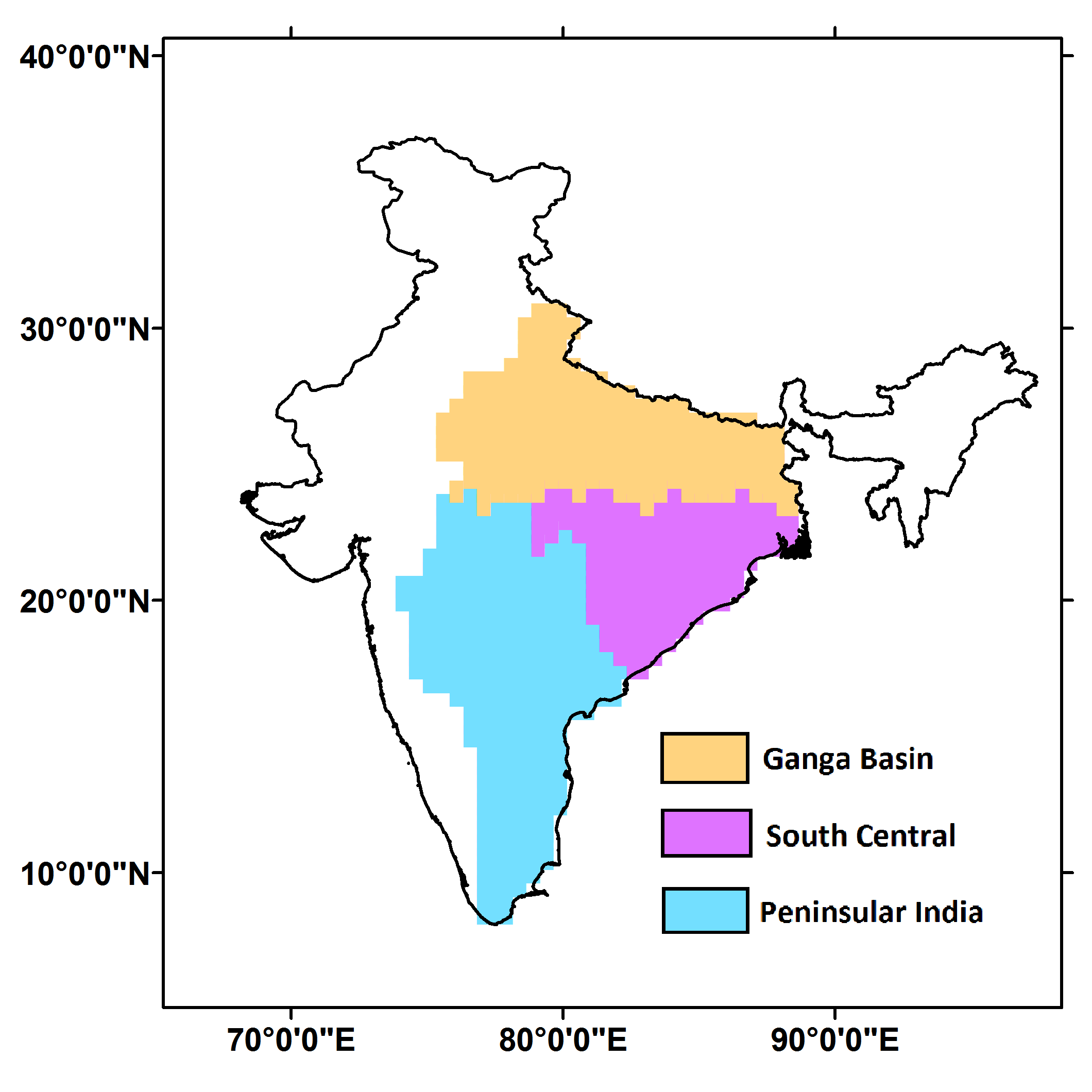


**Figure S8:** Map showing Ganga basin, south central and peninsular Indian regions. The map is generated using ArcGIS 10.1 (http://www.esri.com/software/arcgis).


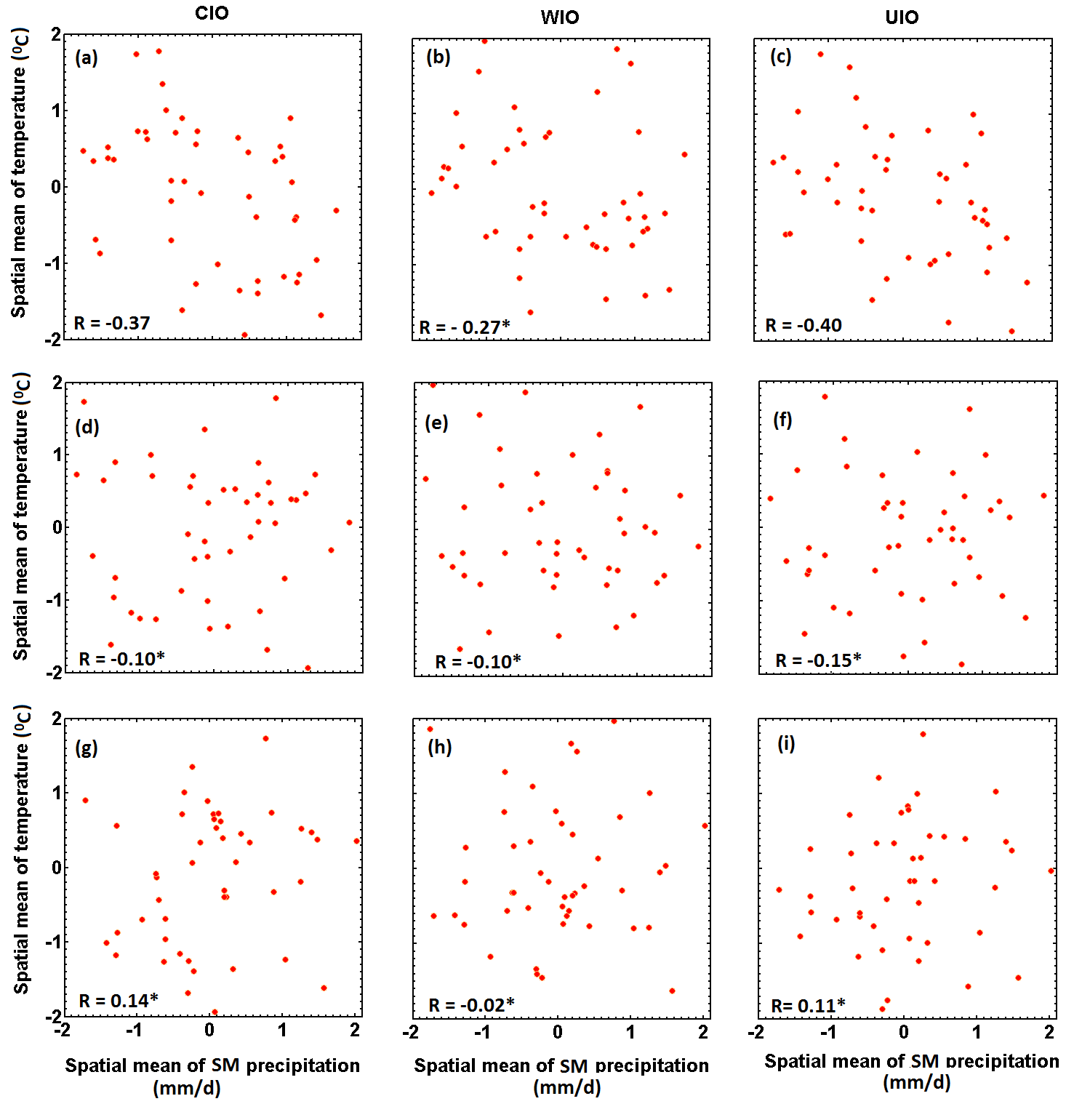


**Figure S9:** Scatter plots of the spatial mean of SST and SM precipitation extremes for different regions considered over India. Top panel (a, b and c) shows the scatter plots for Ganga basin with respect to SSTs over different moisture zones, i.e. CIO, WIO and UIO, respectively. The middle panel (d, e and f) is the same as the top panel, but for south central region, whereas the bottom panel (g, h and f) is for peninsular Indian region. The correlation coefficients are provided in the inset of the individual plots, wherein (*) indicates the correlation which are insignificant at 5% significance level. The figures are developed using MATLAB (http://www.mathworks.com).


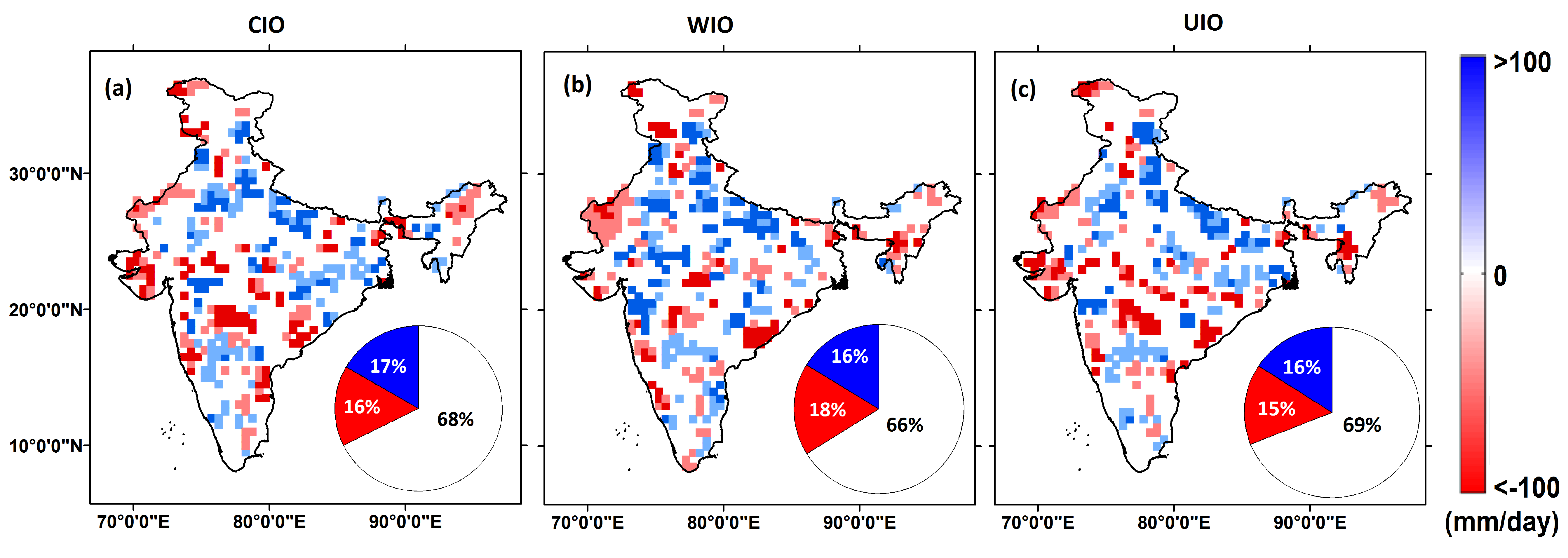


**Figure S10:** Changes in the 50-year RL of SM precipitation intensity from cold to warm years for (a) CIO, (b) WIO and (c) UIO respectively. Following the bootstrap approach, the changes in RL are estimated at the 20% significance level. As shown, 68%, 66% and 69% of the grids show no significant changes in RL from cold to warm years for CIO, WIO and UIO, respectively. The map is generated using ArcGIS 10.1 (http://www.esri.com/software/arcgis).


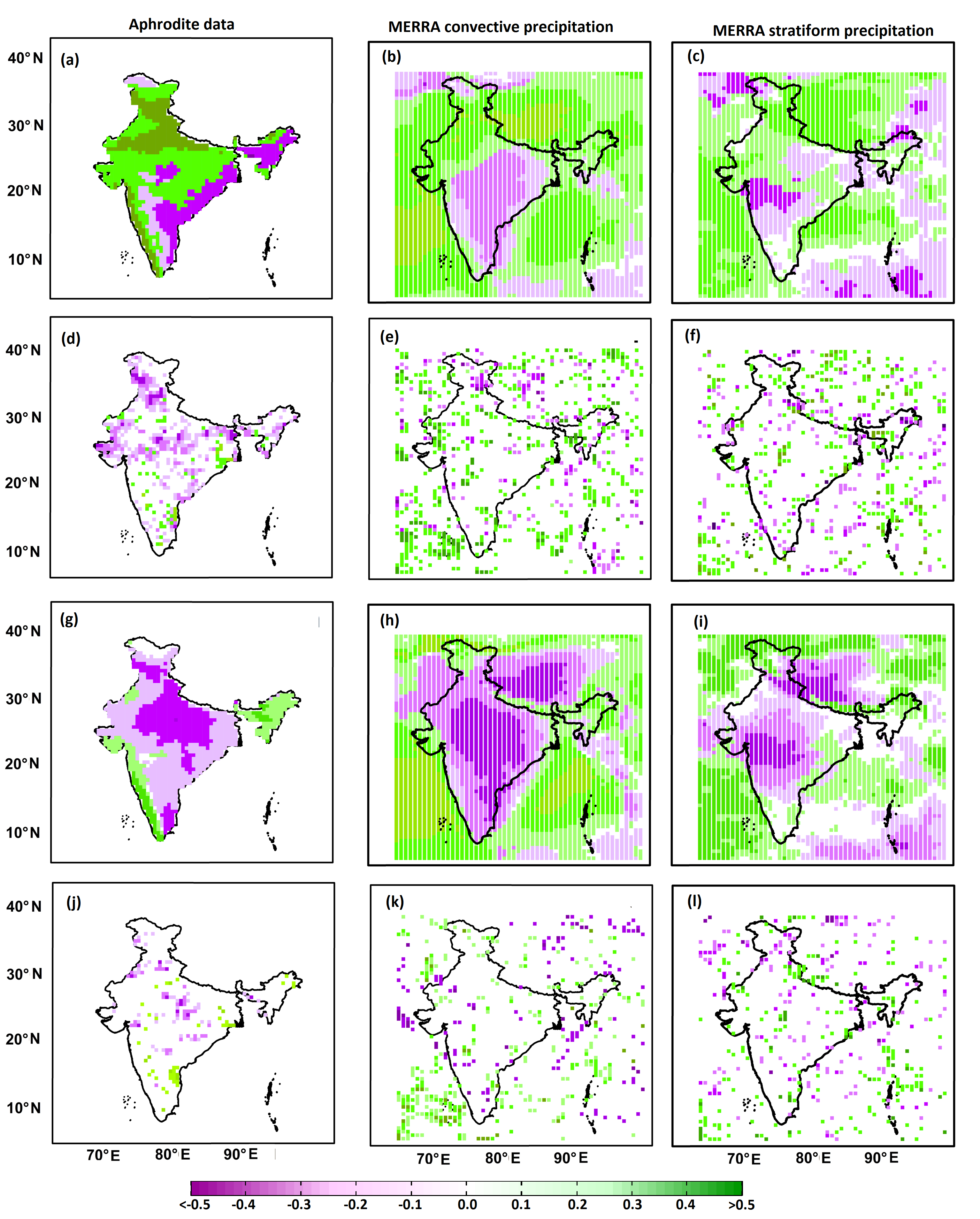


**Figure S11:** Lag correlation analyses between precipitation and temperature. (a) Lag correlation between APHRODITE's total precipitation and 2AT, which shows northern India, is positively correlated with majority of peninsular India exhibiting negative correlation. Similar analysis is performed for MERRA's convective (b) and stratiform precipitation (c). The patterns of correlation are found similar to that of total precipitation. Further, the analysis with extreme rainfall revealed no significant association between 2AT in total (d), convective (e) and stratiform (f) seasonal maxima precipitation. Similar analysis is performed with TIO for both overall (g to i) and extreme precipitation (j to l). The overall precipitation in this scenario is found to be negatively correlated over most of the Indian region, though no significant association is found with extreme precipitation. The map is generated using ArcGIS 10.1 (http://www.esri.com/software/arcgis).

**Table S1:** List of observational data utilized for the study

| **Sl no** | **Data** | **Data duration considered for the study** | **Reference** |
| --- | --- | --- | --- |
| 1 | APHRODITE precipitation data set | 1951-2000 | Yatagai et al. (2005) |
| 2 | CRU-CY temperature data set | 1951-2000 | Harris et al. (2014) |
| 3 | APHRODITE temperature data set | 1982-2000 | Yasutomi et al. (2011) |
| 4 | Monthly SST | 1951-2000 | Rayner et al. (2003) |
| 5 | Daily SST | 1982-2000 | Reynolds et al. (2007) |

**Table S2:** List of GCMs used in the study

| Sl. No. | Name | Institution | Resolution | |
| --- | --- | --- | --- | --- |
|  |  |  | Lat. | Lon. |
| 1 | CanESM2 | Canadian Centre for Climate Modelling and Analysis | 2.7 | 2.8 |
| 2 | EC-Earth | European Earth System Model (ICHEC) | 1.121 | 1.125 |
| 3 | GFDL-ESM2M | Geophysical Fluid Dynamics Laboratory(GFDL) | 2 | 2.5 |
| 4 | MIROC5 | Atmosphere and Ocean Research Institute, National Institute for Environmental Studies, and Japan Agency for Marine-Earth Science and Technology | 1.4 | 1.41 |
| 5 | MPI-ESM-LR | Max-Planck-Inst. For Meteorology | 1.8652 | 1.875 |

**Supplementary References**

Harris, I.P.D.J., Jones, P.D., Osborn, T.J. and Lister, D.H., 2014. Updated high-resolution grids of monthly climatic observations–the CRU TS3. 10 Dataset. International Journal of Climatology, 34(3), pp.623-642.

Rayner, N.A., Parker, D.E., Horton, E.B., Folland, C.K., Alexander, L.V., Rowell, D.P., Kent, E.C. and Kaplan, A., 2003. Global analyses of sea surface temperature, sea ice, and night marine air temperature since the late nineteenth century. Journal of Geophysical Research: Atmospheres,108(D14).

Reynolds, R.W., Smith, T.M., Liu, C., Chelton, D.B., Casey, K.S. and Schlax, M.G., 2007. Daily high-resolution-blended analyses for sea surface temperature. Journal of Climate, 20(22), pp.5473-5496.

Yatagai, A., Kamiguchi, K., Arakawa, O., Hamada, A., Yasutomi, N. and Kitoh, A., 2012. APHRODITE: Constructing a long-term daily gridded precipitation dataset for Asia based on a dense network of rain gauges.Bulletin of the American Meteorological Society, 93(9), pp.1401-1415.

Yasutomi, N., Hamada, A. and Yatagai, A., 2011. Development of a long-term daily gridded temperature dataset and its application to rain/snow discrimination of daily precipitation. Global Environ. Res., 15(2), pp.165-172.
